# A pioneer of modern Chinese Physiology: Dr. Robert Kho-Seng Lim

**DOI:** 10.1007/s13238-019-00655-z

**Published:** 2019-09-05

**Authors:** Fang Zhang

**Affiliations:** China University of Political Science and Law, Beijing, 102249 China

**Figure 1:**
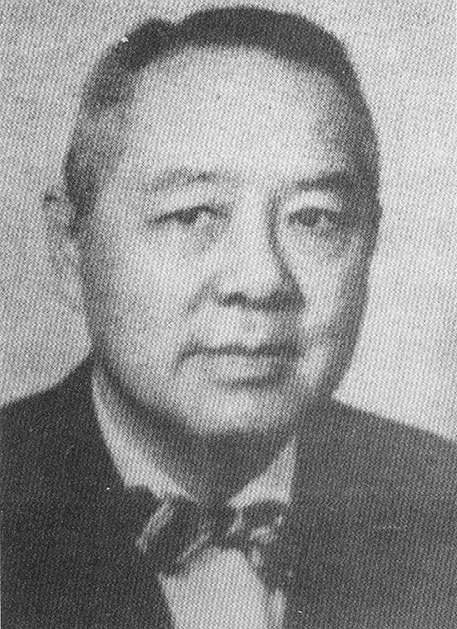
Robert Kho-Seng Lim (1897–1969)

Dr. Robert Kho-Seng Lim (林可胜) was a legendary figure in modern Chinese physiology and medical fields (Fig. 1). He was a famous physiologist and was considered as a pioneer of modern Chinese Physiology. He was also an ardent patriot and organized medical relief corps and trained various medical workers to meet the needs of China during the war. He was a man of modesty, wisdom, fortitude and tenacity.

Dr. Lim was born of Chinese parentage in Singapore on October 15, 1897. His nickname was Booby. His father, Lim Boon-Keng (林文庆), was a distinguished physician graduated from the University of Edinburgh, who promoted social and educational reforms in China. His mother, Wong Tuan-Keng (黄瑞琼), the daughter of Wong Nai-siong (黄乃裳), was one of the first generation of Chinese women who received educations in the United States. His uncle, Wu Lien-Teh (伍连德), was a plague fighter and the father of Chinese public health system.

Dr. Lim went to Scotland when he was eight years old. Later, he attended George Watson’s School in Edinburgh. In 1911, he was admitted to the University of Edinburgh for medical studies after the graduation from Watson’s school. At the outbreak of the First World War, he volunteered and served for the Indian army medical service to train recruits for the field rescue. In 1916, Dr. Lim returned to Edinburgh, where he received his M.B. and Ch.B. degrees in 1919 (Davenport, 1980). In the Medical School, due to his excellent performance, Dr. Lim got appreciated and supported by Sir Edward Sharpey-Schafer, the professor of physiology. After his graduation, he was appointed as lecturer in the department of physiology to teach histology. At the same time, he continued his researches and obtained his Ph.D. degree in 1921. Aged 26, he was elected as fellowship of Royal Society of Edinburgh.

In 1922, Dr. Lim applied for a fellowship from China Medical Board of New York for the opportunities to study in Europe or USA. His application caught attention from Roger S. Greene, the Board’s Secretary. And, he was granted a Rockefeller Foundation Fellowship to work at the University of Chicago for one year.

In 1924, Dr. Lim planned to return to China with his original thought to set up a medical school for Xiamen University. He also planned to work in the Peking Union Medical College (PUMC) for half a year during this transition. At the end, he accepted Greene’s advice and worked as a visiting professor in the PUMC for a year.

At that time, it was relatively lackluster of the department of physiology at PUMC. Upon his arrival, he started to establish a vigorous research program in collaboration with his colleagues and students. Soon, Dr. Lim was appointed as professor of physiology and head of the department at PUMC when he was only 28. Dr. Lim worked in the department of physiology for 12 years from 1924 to 1937 and made outstanding contributions to the development of physiological researches and the construction of the research programs.

Dr. Lim spent all his efforts on team construction, scientific researches, teaching activities, infrastructure construction and talent training for the development of the department of physiology in PUMC. After opening new avenues for the department of physiology in PUMC, Dr. Lim then began to extend the subject of physiology to the whole country. Bring all of the professionals together and carrying out academic exchanges were important ways for the development of physiology in China (Cao, 1998). Dr. Lim and his colleagues founded the Chinese Physiological Society, and Dr. Lim served as the first president of the society. In the following year, Dr. Lim founded *the Chinese Journal of Physiology* and served as editor-in-chief (Luo et al., 2018). Both the Society and the journal had played an essential role in promoting the development of physiology in China.

In addition to the subject construction, Dr. Lim had always been an active scientist in the frontier of physiology, and had around 90 publications. Before the outbreak of the full-scale Anti-Japanese War in 1937, Dr. Lim mainly focused on digestive physiology and circulatory physiology and gained remarkable achievements in both fields.

In the field of digestive physiology, Dr. Lim had successfully conducted various researches on gastric secretion with his colleagues and students with his skilled animal experimental techniques. They had explicitly demonstrated the hormonal mechanism for gastric secretion and the inhibition of gastric motility by fat, which attracted great attentions from scientists all over the world in the field of physiology. In 1930s, he coined the word “enterogastrone” to designate the “gastric inhibitory agent”, which is the first hormone discovered in China (Cao, 1998).

Dr. Lim worked on circulatory physiology from 1936 to 1939 and showed that sympathetic reflexes could be evoked by stimulation of the medulla of the brain. The studies in this research field were temporarily interrupted due to the outbreak of the war. And later on, his colleagues and students continued the researches in this field, which attracted great attentions from the international physiological community.

**Figure 2:**
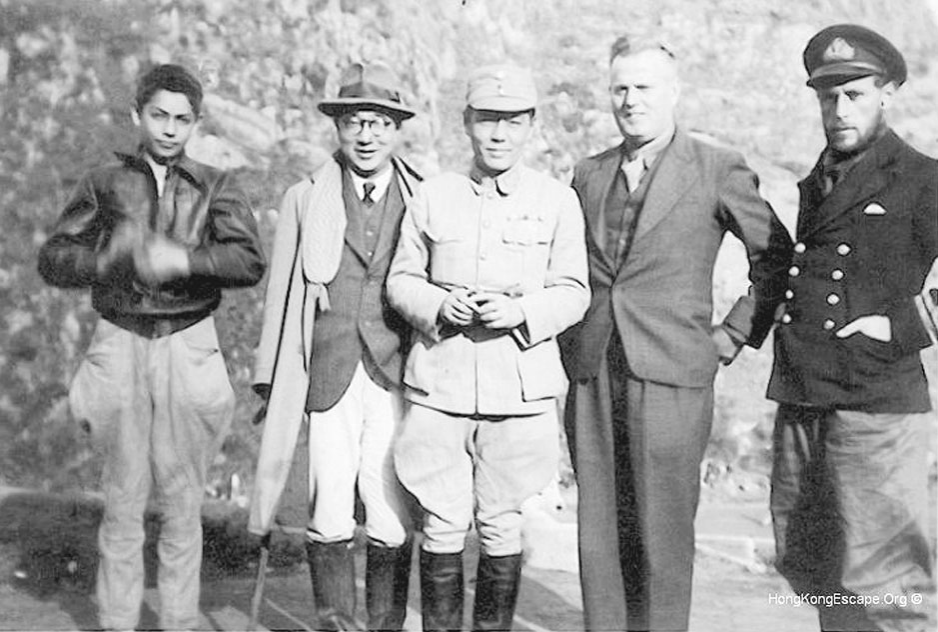
Robert Kho-Seng Lim and others in Kweiyang (in the middle is Dr. Lim)

In the late 1930s, Dr. Lim resigned from his position at PUMC, and started serving his country on a larger scale. As the war began, Dr. Lim founded and directed the Chinese Red Cross Medical Relief Corps, and provided modern medical treatments to the soldiers. He organized the Emergency Medical Service Training School for physicians, nurses, and medical technicians and trained hundreds of medical workers. The graduates served in the army and civilian relief agencies. By 1939, they had led many rescue teams to cover the major battlefields all over the country. Dr. Lim built the largest medical center at Kweiyang (贵阳) in China during the war, and he was appointed as Inspector General of the Medical Services in 1941 (Davenport, 1980) (Fig. 2).

After the war, as Surgeon General, Dr. Lim rebuilt China’s medical education and military medical system, paving the way for the development of military medicine in China. He received the Legion of Merit by President Roosevelt in 1943 and the Medal of Freedom in 1946 given his medical support during the war. He was considered as “one of the greatest men of China”.

In 1948, Dr. Lim was elected to be a member of the Academia Sinica. In the same year, he was offered the position of the Minister of Health, but he rejected this job offer. He went to the United States in 1949 and was invited as a visiting research professor of clinical science at the University of Illinois. From 1950 to 1951, he severed as professor and the head of department of physiology and pharmacology at Creighton University. In 1952, he accepted a position at Miles Laboratories in Indiana, where he was responsible for physiological and pharmacological researches and the guidance of medical scientific research. From his military experience during the war, he became interested in pain relief medications. He found that pain receptors were chemo-sensitive, in which pain-producing agents such as bradykinin peptides were produced when tissues of the body were injured.

Because of his outstanding achievements, Dr. Lim was a member or an honorary member of numerous research institutions and academic societies in the United states, Great Britain and Germany (Ivy, 1970). He was elected a Foreign Associate of the National Academy of Sciences of the United States in 1942. When he became a United States citizen in 1955, he became a regular member of the Academy.

Dr. Lim was diagnosed with esophageal cancer in 1967. In 1969, he moved to his son’s home in Jamaica together with his wife, and his daughter also came back from England. There, he enjoyed his last moments with his family. Dr. Lim passed away on July 8, 1969 at the age of 72.
